# Innovative Strategies to Enhance the Bioavailability of Cannabidiol: Nanotechnology and Advanced Delivery Systems

**DOI:** 10.3390/ph18111637

**Published:** 2025-10-29

**Authors:** Magdalena Paczkowska-Walendowska, Piotr Trzaskoma, Aleksandra Dziopa, Arash Moeini, Michał Soczawa, Zbigniew Krasiński, Judyta Cielecka-Piontek

**Affiliations:** 1 Department of Pharmacognosy and Biomaterials, Poznan University of Medical Sciences, Rokietnicka 3, 60-806 Poznan, Poland; piotrtrzaskoma242@gmail.com (P.T.); jpiontek@ump.edu.pl (J.C.-P.); 2 Department of Pharmacology and Phytochemistry, Institute of Natural Fibres and Medicinal Plants—National Research Institute, Kolejowa 2, 62-064 Plewiska, Poland; aleksandra.dziopa@iwnirz.pl; 3 Chair of Brewing and Beverage Technology, TUM School of Life Sciences, Technical University of Munich, 85354 Freising, Germany; arash.moeini@tum.de; 4 Department of Urology and Urological Oncology, Pomeranian Medical University, Powstańców Wlkp. 72, 70-111 Szczecin, Poland; michal.soczawa@pum.edu.pl; 5 Department of Vascular, Endovascular Surgery, Angiology and Phlebology, Poznan University of Medical Science, Dluga 1/2, 61-848 Poznan, Poland; zkrasinski@ump.edu.pl

**Keywords:** cannabidiol, nanotechnology, drug delivery systems, bioavailability enhancement, nanoemulsions, liposomes, polymeric nanoparticles, cyclodextrin inclusion complexes, personalized medicine

## Abstract

Cannabidiol (CBD), a phytocannabinoid with therapeutic potential for neurological and other conditions, faces significant challenges in bioavailability due to its low water solubility, high lipophilicity, and extensive first-pass metabolism. Researchers have developed advanced nanodelivery systems addressing these limitations to enhance CBD’s absorption, stability, and efficacy. This review provides not only a comprehensive summary of current nanotechnological delivery strategies for CBD, including nanoemulsions, liposomes, polymeric micelles, nanosuspensions, and cyclodextrin inclusion complexes, but also introduces a distinct comparative and integrative perspective. Unlike previous reviews, our work synthesizes preclinical and clinical evidence while highlighting the novel integration of nanotechnology with bioenhancers and personalized medicine approaches. We further emphasize the emerging concepts of hybrid and smart nanocarriers, which have not yet been systematically discussed, positioning them as next-generation solutions to overcome CBD’s bioavailability challenges and paving the way for precision therapeutics.

## 1. Introduction

One of the most notable trends in modern medicine is the increasing use of phytocannabinoids for treating neurological conditions [1]. Among them, cannabidiol (CBD) stands out as a non-psychoactive compound, contrasting with Δ^9^-tetrahydrocannabinol (THC). Interestingly, CBD can even counteract the effects of cannabinoid intoxication [2]. Despite its numerous benefits, the biggest challenge in using CBD remains its poor bioavailability, which is primarily due to low absorption in the digestive system and rapid metabolism in the liver [3].

Currently, the most common methods of CBD consumption include vaporization (e.g., dried hemp) and sublingual administration (CBD oils) [4]. In some countries, CBD-containing prescription drugs can also be formulated into ointments or suppositories by pharmacists. However, challenges such as throat irritation from vaporization or incorrect ingestion of sublingual formulations highlight the need for better oral delivery methods that ensure efficient absorption. Although several recent reviews have summarized strategies to enhance CBD bioavailability [5,6,7,8], there is still a lack of integrative analyses that directly compare nanocarrier systems, highlight their translational readiness, and explore future directions such as hybrid and smart nanocarriers. In particular, the field lacks a systematic discussion of how nanotechnology can be combined with bioenhancers and personalized medicine approaches to overcome metabolic and pharmacokinetic barriers. Therefore, the central question guiding this review is: which advanced delivery systems hold the greatest promise for translating CBD into clinically effective and personalized therapeutics? By addressing this gap, our work provides a comparative and forward-looking perspective that distinguishes it from earlier reviews.

### 1.1. Cannabidiol (CBD)

Cannabidiol is a phytocannabinoid naturally found in hemp plants, specifically *Cannabis sativa* L. and *Cannabis indica* Lam. [9]. Its pure form is a highly lipophilic white powder (logP = 6.6). Hemp is the only species in the *Cannabaceae* family known to produce cannabinoids. Recently, CBD-based products have gained popularity, appearing in dietary supplements, on pharmacy shelves, and in specialized hemp stores. The medical sector has also introduced prescription CBD medications, including Epidiolex, a 100 mg/mL CBD solution in sesame oil, and Sativex, an alcoholic solution containing both CBD and THC [10]. These drugs are approved for treating conditions such as seizures associated with Lennox–Gastaut syndrome, Dravet syndrome, and tuberous sclerosis, as well as spasticity in multiple sclerosis [11].

However, emerging research suggests that CBD may have broader therapeutic potential, including applications in treating mental illness, anxiety, diabetes, and pain [12]. Although its exact mechanism of action is not fully understood, CBD is believed to act as an antagonistic modulator of the G-protein-coupled cannabinoid CB1 receptor (GPCR) [13]. Some studies also suggest that CBD interacts with other receptors, including G protein-coupled receptor 12 and 55 (GPR12, GPR55), glycine receptor alpha-1, alpha-3, and beta (GlyRα1, GlyRα3, GlyRβ), 5-hydroxytryptamine receptor 1A, 2A, and 3A (5-HT1A, 5-HT2A, 5-HT3A), peroxisome proliferator-activated receptor gamma (PPARγ), and the adenosine A1 receptor (ARA1) (Table 1) [14]. These interactions may contribute to its diverse therapeutic effects, which continue to be the subject of extensive scientific investigation.

### 1.2. Bioavailability and Bioefficacy

Although CBD is widely used for treating nervous-system-related ailments, it does have its limitations. According to the biopharmaceutical classification system, CBD is classified as a class 2 drug, meaning it has negligible water solubility (12.6 mg/L) but high permeability [28]. Additionally, CBD undergoes a strong first-pass effect, which eliminates approximately 75% of the absorbed dose, further reducing its effectiveness [29]. As a result of these factors, the oral bioavailability of CBD is extremely low, at only 6%.

One way to improve CBD absorption is by consuming it with a high-fat meal that is FDA/EMA-approved, which can increase bioavailability by up to four times. Studies have shown that when a 1500 mg dose of CBD was taken with a high-fat meal, the highest concentration (1628 ng/mL) was reached within three hours [30]. Given these challenges, poor bioavailability and rapid metabolism, there is a growing need for alternative drug delivery methods to enhance absorption while reducing hepatic metabolism. Such approaches could lead to faster onset and prolonged therapeutic effects, making CBD treatment more effective.

## 2. Methodology of Literature Review

A systematic literature review was conducted to provide a comprehensive overview of current nanotechnological strategies aimed at improving the bioavailability of CBD. The search was performed across three major scientific databases: PubMed, Scopus, and Web of Science, covering the period from 2010 to 2025. Only peer-reviewed articles published in English were considered for inclusion. The primary search terms included: (cannabidiol OR CBD) AND (nanotechnology OR nanocarriers OR nanoemulsions OR liposomes OR polymeric nanoparticles OR micelles OR cyclodextrins OR bioavailability OR drug delivery). The keywords were applied to titles, abstracts, and author-defined keywords, and searched both individually and in combination to capture all relevant variations and formulations used in the literature.

The following inclusion criteria were applied: (1) Original research papers or review articles focusing on the application of nanotechnology or advanced delivery systems for CBD; (2) Studies investigating the enhancement of CBD solubility, stability, permeability, or overall bioavailability; (3) Experimental, preclinical, or clinical studies that evaluated formulation performance or pharmacokinetic outcomes.

The exclusion criteria were as follows: (1) Publications not directly related to pharmaceutical or biomedical applications of CBD; (2) Studies concerning cannabinoids other than CBD; (3) Reports limited to macro-scale or non-nanotechnological approaches; (4) Conference abstracts, patents, incomplete reports, or sources lacking full data; (5) Papers addressing biological effects of CBD unrelated to its delivery or bioavailability (e.g., receptor pharmacodynamics or behavioral studies).

After applying these criteria, 58 articles were deemed eligible for full-text analysis and included in the final review. Additionally, the reference lists of key reviews and experimental papers were manually examined to identify any relevant studies not captured during the initial database search. The remaining works cited in the paper concern general information about CBD and basic information about formulation types.

## 3. Lipid-Based Nanocarriers

### 3.1. Nanoemulsions

A nanoemulsion is a thermodynamically stable formulation consisting of two immiscible liquids combined into a single phase using emulsifying agents. The particle sizes in nanoemulsions range from 10 to 1000 nm and are typically spherical. This structure significantly enhances active ingredient absorption by increasing the emulsion droplets’ surface area [31]. Nanoemulsions are created using various oils (such as castor oil, corn oil, coconut oil, evening primrose oil, linseed oil, mineral oil, olive oil, and peanut oil) and emulsifying agents, including spans, tweens, hydrophilic colloids (such as xanthan gum, acacia gum, and guar), and finely divided solids (such as bentonite and veegum) [31]. Researchers have leveraged the properties of nanoemulsions to develop CBD-containing formulations aimed at improving their bioavailability.

Nakano et al. formulated CBD nanoemulsions (CBD-NEs) using water as the continuous phase, along with a 1.7:3.8:70 (*w*/*w*%) mixture of Vitamin E acetate, ethanol, and Tween-20 as the oil phase, surfactant, and co-surfactant, respectively [32]. The resulting emulsion contained CBD at a concentration of 30 mg/mL, with an initial droplet size of 35.3 ± 11.8 nm, which increased to 44.5 ± 14.0 nm after one week. In another formulation with a 5 mg/g CBD concentration, the droplet size was initially 102 nm, increasing to 389 nm over time. The CBD-NE formulation exhibited 1.65 times higher bioavailability than standard CBD oil in Wistar rats (*p* < 0.001) [32]. Additionally, researchers reported a bioavailability increase of 93.9% compared to the 73.3% bioavailability obtained in a previous study by Zgair et al., representing a 1.28-fold improvement [33]. The formulation also demonstrated a 3.3-fold lower T_max_ than conventional CBD oil (*p* < 0.001) [32], meaning it reached peak concentration in the bloodstream significantly faster. This property is particularly beneficial for treating pain, anxiety, or epileptic seizures, where rapid onset of action is essential.

Although oral nanoemulsions have shown enhanced bioavailability compared to traditional CBD oil, they remain less popular due to issues with physical instability (such as phase separation, droplet growth, or Ostwald ripening, which can compromise long-term storage and reproducibility), complex and costly manufacturing (requires high-energy input, e.g., ultrasonication or high-pressure homogenization), potential safety concerns related to surfactants, and regulatory challenges regarding reproducibility and large-scale production.

Therefore, researchers have increasingly utilized the Self-Nanoemulsifying Drug Delivery System (SNEDDS) to simplify the formation of CBD-containing nanoemulsions. SNEDDS contains the same ingredients as an emulsion but lacks an aqueous phase in its composition. Instead, when it meets an aqueous environment, such as gastric fluid, it spontaneously forms a nano- or micro-emulsion [34]. SNEDDS technology is widely used to enhance the solubility and permeability of poorly water-soluble drugs, with several formulations already available on the market, including isotretinoin, cyclosporine A, ritonavir, and testosterone undecanoate. Among the various nanoemulsion formulations studied, SNEDDS is the most frequently cited as having a high potential for increasing CBD bioavailability [35].

Using MCT oil, mixed surfactants (Labrasol, Gatteffosse, Lyon, France; Tween 80, not-further defined), and Transcutol (Gatteffosse, Lyon, France) as a co-surfactant, optimized SNEDDSs were prepared by Wu et al. and subsequently converted into solid powders via carrier adsorption and spray drying. The solid SNEDDS (S-SNNEDS) demonstrated improved solubility, rapid self-emulsification, favorable physicochemical stability, and enhanced in vitro release compared with conventional oil-based formulations. In vivo pharmacokinetic studies in rats further confirmed the superior absorption and higher C_max_ for S-SNEDDS (287.7 ng/mL; *p* < 0.05) to CBD–sesame oil (186.2 ng/mL) compared to CBD–MCT (13.4 ng/mL) [36]. In another study, a polyglycerol (PG)-based SEDDS showed superior mucus permeability and enhanced lipolysis compared to PEG-based counterparts, attributable to favorable surface properties and higher fatty acid release. In vivo pharmacokinetic evaluation revealed that the PG-based SEDDS achieved improved absolute bioavailability (3.8%) relative to Epidiolex® (Jazz Pharmaceuticals, Dublin, Ireland) (3.4%), with all SEDDS formulations reaching higher maximum plasma concentrations (30.6–35.8 ng/mL) than Epidiolex (25.0 ng/mL). These results highlight the potential of PG-based SEDDSs as a promising strategy to enhance the solubility, permeability, and systemic exposure of orally administered CBD [37].

A clinical study conducted by Izgelov et al. investigated CBD–SNEDDS in a three-way, blinded, crossover study involving 12 healthy male volunteers [38]. CBD was administered in three forms: pure CBD powder, dissolved in sesame oil (CBD–sesame oil), and CBD–SNEDDS. The SNEDDS formulation was prepared by dissolving CBD in a pre-mixed oil phase containing ethanol, soy lecithin, sesame oil, and surfactants such as Tween 20, Span 80, and Kolliphor RH40, resulting in a final CBD concentration of 10%. To test the formation of the nanoemulsion, the formulation was vortex-mixed with simulated gastric fluid (pH 1.2) in a 1:50 dilution. The resulting nanoemulsion droplets had an average diameter of 39 ± 8 nm. The study found that CBD–SNEDDS significantly increased bioavailability. Compared to pure CBD powder, the C_max_ increased 22-fold, while the AUC increased 7-fold. Compared to CBD–sesame oil, CBD–SNEDDS resulted in a 17-fold higher C_max_ and an 8-fold higher AUC. Participants reported drowsiness as the most common side effect, along with moderate bloating and abdominal pain, which did not require medical intervention. While various formulations have successfully increased CBD bioavailability, they have not effectively reduced the first-pass metabolism of the drug. To address this issue, researchers developed Advanced Pro-Nanolipospheres (PNLs), which function similarly to SNEDDSs but include additional components inhibiting phase I and phase II metabolism, enhancing drug absorption. These ingredients often include active components from plant extracts, such as alkaloids or polyphenols.

In the next study, two participants received either a buccal dose of Sativex (10 mg CBD) or P-PNL–CBD (10 mg CBD with 20 mg Piperine). The P-PNL–CBD group showed a 4-fold increase in C_max_ and a 2.2-fold increase in AUC compared to Sativex. However, while this formulation significantly improved CBD absorption, participants reported somnolence as the most common side effect, along with anxiety, dizziness, hand numbness, nausea, reflux, and vomiting [39]. Despite its promising results, the P-PNL–CBD formulation may pose risks of drug interactions, as it can alter the metabolism of other medications. This could lead to toxic drug concentrations in the bloodstream, making dose adjustments necessary when using P-PNL–CBD alongside other chronic medications. Therefore, careful monitoring and dosage modifications may be required to ensure safe and effective use.

SNEDDSs markedly improve CBD solubility, dissolution, and intestinal absorption, resulting in higher C_max_ and AUC compared to oil-based formulations, while their spontaneous self-emulsification simplifies preparation and ensures reproducibility. Nevertheless, they do not fully overcome first-pass metabolism, require high surfactant concentrations that may cause irritation, and often need conversion into solid forms for stability, which increases production complexity and cost. Moreover, advanced variants such as Pro-Nanolipospheres further enhance absorption but pose risks of drug–drug interactions. Thus, despite their promise, the safety, scalability, and metabolic limitations of CBD–SNEDDSs restrict their widespread application.

### 3.2. Nanoliposomes

Liposomes are spherical vesicles composed of one or more concentric lipid bilayers surrounding a water-based core. Thanks to their lipophilic (lipid envelope) and hydrophilic (water core) structure, liposomes can effectively deliver fat- and water-soluble substances [40]. Their properties can vary based on size, number of layers, lipid composition, and surface charge, which influence drug loading capacity, permeability, stability, and release profile. By modifying these parameters, liposomes can be tailored for specific drug delivery applications, including enhancing absorption through biological membranes, increasing drug stability, and controlling the release of active ingredients.

Liposomes are classified based on their size into different categories: Multilamellar Vesicles (MLVs; >500 nm), Multivesicular Vesicles (MVVs; >1000 nm), Giant Unilamellar Vesicles (GUVs; >1000 nm), Large Unilamellar Vesicles (LUVs; >100 nm), and Small Unilamellar Vesicles (SUVs; 20–100 nm) [41]. They can also be categorized by composition and function, including Ethosomes (which contain 25–40% ethanol to promote absorption), Binary Ethosomes (which include additional alcohols like propylene glycol for enhanced stability), Transferosomes (capable of deformation for increased permeability) [42], Polymerosomes (made of synthetic copolymers for increased flexibility and permeability), Niosomes (containing non-ionic surfactants), and many other formulations designed to improve stability and bioavailability [43].

A study by Franzè et al. developed a CBD-loaded liposomal transdermal system to enhance skin penetration [44]. The formulations consisted of soybean phosphatidylcholine (SPC), Tween 80 (in some formulations), and a buffered core at pH 2.5 (with a pH 6.5 dispersant medium). The CBD encapsulation efficiency was 63.7 ± 4.1% for liposomes containing Tween 80 and 76.0 ± 1.4% for those without it, with particle sizes of 107.1 ± 0.7 nm and 103.0 ± 0.2 nm, respectively. A 1% CBD solution in PEG 400 was used as a reference. Skin permeation tests, conducted using a modified Franz diffusion cell and porcine ear skin, demonstrated that liposomes without Tween 80 doubled CBD permeability, while liposomes with Tween 80 enhanced permeability by four times compared to the reference CBD solution. However, the study also noted that the presence of Tween 80 might reduce CBD absorption, suggesting the need for further investigation to prevent potential inhibition of drug uptake [44].

Further research by Fu et al. explored modified liposomes containing CBD, 20(S)-protopanaxadiol (PPD, used as a cholesterol substitute), and *n*-Dodecyl β-D-maltoside (Mal, used as a surfactant) [45]. Two types of liposomes were formulated: CP-liposomes (containing SPC, PPD, and CBD in a 4:1:1 mass ratio) and GMCP-liposomes (containing SPC, PPD, CBD, and Mal in a 4:1:1:1 ratio). The encapsulation efficiency for CBD was nearly 100%, with CBD concentrations of 14.26% in CP-liposomes and 12.17% in GMCP-liposomes. The average particle sizes were 138.8 nm for CP-liposomes and 179.3 nm for GMCP-liposomes, demonstrating the effectiveness of these modified liposomal formulations in incorporating CBD while maintaining high stability and drug entrapment efficiency. The permeation efficacy of CBD was evaluated in vitro on various cancer cell lines, including 4T1, MCF-7, A549, C6, HeLa, and HepG2, by measuring tumor size reduction. The study found that both CP-liposomes and GMCP-liposomes effectively decreased tumor size, demonstrating their potential for enhanced CBD delivery in cancer treatment. As shown in Table 2, both formulations exhibited a lower IC_50_ than the control sample containing free CBD, suggesting that the increased permeability of the liposomal formulations contributed to their greater efficacy. Although the results for CP-liposomes and GMCP-liposomes were not significantly different, CP-liposomes demonstrated slightly better performance in vitro. This indicates that while both formulations effectively enhance CBD absorption and anti-cancer activity, CP-liposomes may offer a slight advantage regarding permeability and tumor reduction [45].

Another study by Moqejwa et al. explored the use of CBD-loaded transferosomes to enhance bioavailability through rectal administration [46]. The transferosomes were formulated using 60 mg soy lecithin, 100 mg polysorbate 80, and 30 mg cholesterol, with PBS buffer as the aqueous phase. The resulting nanoparticles had an average size of 87.31 ± 12.62 nm and demonstrated 80 ± 0.077% entrapment efficiency. To assess ex vivo permeability, the researchers used a Franz diffusion cell with colorectal tissue from Sprague Dawley rats. The findings revealed that CBD isolate alone permeated the tissue at a rate of 1 mg/cm^2^/h, whereas CBD in transferosomes permeated at 1.7 mg/cm^2^/h, indicating a 70% increase in bioavailability (*p* < 0.05). The surfactants in the transferosomes provided greater flexibility, allowing deeper tissue penetration and drug release at the target site. The formulation exhibited a rapid release profile, delivering 95% of the dose within 7 h, making it a promising alternative delivery system for CBD [46].

Liposomes improve CBD solubility, stability, and permeability, offering controlled release and the ability to cross biological membranes, with studies showing enhanced skin penetration and antitumor activity. However, they face notable drawbacks, including physical and chemical instability that can lead to CBD leakage, vesicle fusion, or lipid oxidation during storage. Their production often requires costly techniques and strict conditions to maintain uniformity, which complicates large-scale manufacturing. Surfactants and additives may unpredictably affect CBD absorption or cause local irritation. Furthermore, most evidence for CBD-loaded liposomes is limited to preclinical or in vitro studies, and translational readiness remains uncertain. Compared to other nanocarriers, liposomes may face greater regulatory scrutiny due to challenges in reproducibility, stability, and safety upon long-term administration.

### 3.3. Nanosuspension

Suspensions are a well-known drug formulation commonly used for antacids, antibiotics, glucocorticosteroids, analgesics, and anthelmintics. This form consists of a heterogeneous mixture containing solid drug particles that are poorly dispersible in a solvent. Typically, suspension particles measure below 1 µm, while nanosuspensions have particle sizes below 500 nm, allowing for parenteral administration, unlike classical suspensions [47].

A study by Fu et al. investigated CBD nanosuspensions to enhance bioavailability through intramuscular administration [48]. The researchers used antisolvent precipitation, employing Tween 80 (0.50% *w*/*v*) as an antisolvent, to produce CBD nanocrystals measuring 141.7 ± 1.5 nm. These were then lyophilized with bovine serum albumin as a cryoprotectant. In in vitro dissolution studies, the raw CBD material exhibited a 42.91% cumulative dissolution rate, whereas the nanosuspension formulation achieved 91.57% cumulative solubility. The in vivo study, conducted on a rat model, compared oral and intramuscular administration of the nanosuspension with an orally administered conventional CBD oil formulation. The results demonstrated that intramuscular and oral nanosuspension formulations reached higher C_max_ values than oral CBD oil. The C_max_ values for intramuscular, oral nanosuspension, and oral commercial oil formulation were 239.41 ± 16.92 (*p* < 0.05 compared to IV values), 151.40 ± 35.78, and 135.94 ± 38.15 ng/mL, respectively [48].

Another study by Caggiano et al. explored CBD nanosuspensions using flash nanoprecipitation [49]. The researchers stabilized the formulation using lecithin or hydroxypropylmethylcellulose acetate succinate (HPMCAS) and incorporated Fe_3_O_4_ for co-encapsulation. The ingredients were mixed in a 1:1:1 mass ratio (CBD/stabilizer/Fe_3_O_4_), yielding nanoparticles with sizes of 156 ± 10 nm (CBD/lecithin/Fe_3_O_4_) and 287 ± 11 nm (CBD/HPMCAS/Fe_3_O_4_). Both formulations were tested for dissolution in a simulated intestinal medium, revealing a 6-fold increase in solubility compared to crystalline CBD. Additionally, the lecithin-based formula exhibited an immediate and complete burst release. In contrast, the HPMCAS formulation showed an initial rapid release followed by a gradual increase, indicating different drug release profiles depending on the stabilizer used [49].

### 3.4. Oleosomes

Oleosomes are specialized plant organelles primarily responsible for storing neutral lipids, mainly triacylglycerols (TAGs), which represent the main energy reserve for germinating seeds [50]. Their diameter typically ranges from 0.2 to 2 µm, although in some species, larger structures reaching several micrometers may also occur [51]. The interior of the oleosome is filled with a hydrophobic triacylglycerol core surrounded by a phospholipid monolayer, which distinguishes them from most other cellular organelles enclosed by a bilayer membrane. Owing to their specific structure and the presence of surface proteins, oleosomes function as natural oil–water emulsions that remain stable without the need for additional emulsifiers [50]. Their structural durability allows them to maintain integrity even under seed desiccation and during long-term storage [51].

The potential of oleosomes as carriers for lipophilic compounds was confirmed in a study by Ji et al., which compared three formulations of cannabidiol (CBD)—rapeseed oil, an artificial oil–water emulsion, and CBD-loaded rapeseed oleosomes. Oleosomes demonstrated the highest level of in vitro lipolysis (68.3%) compared to the emulsion (36.2%) and oil (16.8%), facilitating CBD incorporation into micelles (~90%). In in vivo studies in rats, oleosomes yielded significantly improved pharmacokinetic parameters: C_max_ reached 79 ng/mL versus 36 ng/mL (oil) and 50 ng/mL (emulsion), while total bioavailability (AUC_0–∞_) was 413 h·ng/mL, nearly double that of oil (*p* < 0.001). Most notably, lymphatic transport was markedly enhanced—CBD concentrations in mesenteric lymph nodes were 8.5-fold higher than with oil, and in lymph fluid even 26.5-fold greater [52]. The authors emphasize that, due to the combination of a phospholipid monolayer and surface proteins, oleosomes represent a stable and highly efficient delivery system for bioactive compounds, surpassing conventional emulsions.

Oleosomes are natural, biocompatible carriers that enhance CBD solubility, bioavailability, and lymphatic transport while remaining stable without synthetic emulsifiers. However, their large-scale application is limited by variability in plant-derived sources, challenges in standardization, and the lack of clinical validation, raising concerns about reproducibility and regulatory acceptance.

## 4. Polymeric/Biopolymeric Nanocarriers

### 4.1. Polymeric Micelles

Polymeric micelles are amphiphilic nanoparticles with a spherical structure composed of two main parts: a hydrophobic core and a hydrophilic shell. These micelles typically range in size from 5 to 100 nm. Each part plays a crucial role in drug delivery—the core serves as a reservoir for lipophilic drugs, while the outer hydrophilic shell stabilizes the core and enhances water solubility [53].

A research group led by Liu et al. explored CBD-loaded nanomicelles for inflammation-targeted drug delivery [54]. To prepare these micelles, CBD and a synthesized fucoidan ester with deoxycholic acid (FD) were dissolved in methanol, which was later evaporated after mixing with PBS. The resulting micelles measured 118.75 ± 1.46 nm, with an encapsulation efficiency of 88.8 ± 0.2%. A release study showed that these micelles exhibited prolonged drug activity compared to free CBD. Additionally, the formulation demonstrated faster drug release in lysosomal pH conditions (pH 5.0) due to ester bond hydrolysis, suggesting that CBD would be efficiently released upon cellular internalization. The therapeutic potential of CBD/FD micelles was evaluated in mice by observing ulcerated areas of the tongue. The rodents received the drug either intravenously or in situ as free CBD or CBD/FD micelles. The results revealed that CBD/FD micelles administered in situ significantly reduced ulceration compared to intravenous and in situ administration of free CBD [54].

Another study by Su et al. focused on polymeric micelles composed of Poloxamer 407 and rubusoside [55]. These micelles were formed via self-assembling these components, followed by solvent evaporation for stabilization. The resulting micelles had an average size of 103 ± 2.66 nm and exhibited an impressive encapsulation efficiency of 92.8% ± 4.7%. The stability of these micelles was tested over 6 months at different temperatures, showing no significant degradation or loss of properties. Moreover, this formulation significantly improved CBD’s water solubility, reaching 15.60 mg/mL, representing a 1560-fold increase compared to its natural solubility [55].

### 4.2. Polymeric Nanoparticles

Polymeric nanoparticles include nanocapsules and nanospheres, which can encapsulate active compounds entrapped within or adsorbed onto the polymeric core. These particles typically range in size from 1 to 1000 nm. Increasingly, polymeric nanoparticles are being recognized for their potential in targeted drug delivery, offering benefits such as controlled release, enhanced stability, and improved bioavailability of drugs [56].

A study by Fraguas-Sánchez et al. explored using polylactic-*co*-glycolic acid (PLGA) nanoparticles to enhance CBD bioavailability [57]. The CBD-loaded PLGA nanoparticles (CBD-NPs) were prepared using the emulsion solvent evaporation technique, where CBD was incorporated into 100 mg of PLGA to achieve a concentration of 1.5% (*w*/*w*). The resulting particles measured 236 ± 12 nm and showed an encapsulation efficiency of 95.23% ± 3.30%, indicating nearly 100% drug loading. The formulation displayed a high burst effect, with 35% CBD released within the first hour, followed by prolonged release over 96 h. This profile suggests the formulation could provide a rapid onset of action with extended duration. In an in vitro study, SKOV-3 ovarian cancer cells were treated with CBD-NPs, free CBD (CBDsol), and PLGA nanoparticles without CBD (PLGA-NPs) at increasing concentrations (5 to 50 µM) for 24 to 48 h. The results showed that CBD-NPs demonstrated enhanced antiproliferative activity compared to free CBD. The IC_50_ values (concentration required to inhibit cell proliferation by 50%) after 24 h were 33.19 ± 2.57 µM for CBD_sol_ and 29.64 ± 2.94 µM for CBD-NPs. After 48 h, the IC_50_ for CBD_sol_ was 23.47 ± 4.10 µM, while CBD-NPs showed a lower IC_50_ of 20.88 ± 1.25 µM. These results suggest that CBD nanoencapsulation enhances its anti-cancer efficacy, particularly at lower concentrations. The CAM (chicken embryo membrane) model was also used for testing on tumorigenic SKOV-3 tumors. The CBD-NPs formulation demonstrated slightly better tumor growth inhibition than free CBD (CBD_sol_), with a 1.5-fold greater inhibition than 1.38-fold inhibition by CBD_sol_ [57].

In another study, Tran et al. and Wang et al. investigated the use of zein (a protein from corn) and whey protein (WP) to create polymeric nanoparticles [58,59]. Traditionally used in coatings, zein was explored here as a drug delivery material in combination with WP, which can form an interpolymer complex. These nanoparticles were designed to improve the solubility and bioavailability of CBD upon oral administration. CBD was dissolved in a zein solution (25 mg/mL in 90% ethanol) to a 1 mg/mL concentration and then mixed with WP to create these nanoparticles. The solvent was evaporated, and the resulting dry product was dissolved in water, with large precipitates removed. The resulting nanoparticles had sizes ranging from 140 to 160 nm, depending on the WP concentration, with an encapsulation efficiency of up to 89%. An API solubility test revealed a remarkable improvement in CBD solubility from 0.39 µg/mL to a range of 184–200 µg/mL for the nanoparticle formulations (*p* < 0.05), resulting in a 465–505-fold increase in solubility. An in vivo bioavailability study was conducted on male Sprague Dawley rats, administering both the zein–WP nanoparticles and pure CBD at 40 mg/kg. The results showed that the CBD nanoparticles achieved significantly higher concentrations than pure CBD. For example, C_max_ was 0.232 µg/mL for pure CBD, while the nanoparticles yielded 0.466 µg/mL, a 2-fold increase. Similarly, the AUC for CBD in the nanoparticles was 2.912 µg/mL/h, compared to 1.657 µg/mL/h for pure CBD, a 1.75-fold increase. The authors suggested that the increased bioavailability of CBD from the nanoparticles could result from several factors, including better dispersion of the API in water, mucoadhesive properties of zein, stability against gastric enzymes like pepsin, resistance to pancreatic degradation, and particle size that facilitates endocytosis by epithelial cells [59].

Polymeric nanocarriers improve CBD solubility, stability, absorption, and controlled release, showing superior pharmacological effects compared to free CBD; however, their translation is limited by several factors. Many rely on synthetic or semi-synthetic polymers that may raise safety and biodegradability concerns, especially with chronic use, while some exhibit burst release effects that compromise controlled dosing. Complex manufacturing techniques and solvent use hinder scalability and reproducibility, and protein-based carriers (e.g., zein, whey protein) may suffer from variability in raw material quality. Moreover, most evidence remains confined to in vitro or animal models with little clinical validation, and regulatory acceptance is further challenged by safety testing requirements for novel excipients and the lack of standardized production protocols.

## 5. Cyclodextrin Inclusion Complex

Cyclodextrins are a family of cyclic oligosaccharides with a hydrophilic outer surface and a lipophilic inner cavity. These large molecules contain numerous hydrogen bond donors and acceptors, forming a “molecular cage”. Due to their structure, cyclodextrins can significantly enhance the aqueous solubility of poorly soluble substances, improving both bioavailability and stability. A key feature of natural α- and β-cyclodextrins is that human salivary and pancreatic amylases do not digest them but can be metabolized by intestinal microflora. This property allows cyclodextrin-based formulations to bypass the stomach and deliver the active pharmaceutical ingredient (API) to the intestine, where absorption occurs [60]. The main difference between α- and β-cyclodextrin is their solubility in water at 25 °C, which is approximately 12–14 g/100 mL for α-CD, while β-CD has a solubility of only approximately 1.8 g/100 mL [61]. The lower solubility of β-CD results from the presence of an internal band of hydrogen bonds formed between the hydroxyl groups of adjacent glucopyranose units [62], which limits its ability to interact with water molecules. Additionally, both forms differ in the diameter of the central cavity, which for α-CD is approximately 0.57 nm and for β-CD is approximately 0.78 nm [63]. This difference determines the complexing preferences—the larger diameter of the β-CD cavity favors the formation of inclusion complexes with larger molecules and a more complex spatial structure [64].

A study by Li et al. explored the potential of cyclodextrins to improve the solubility of CBD by creating an inclusion complex with β-cyclodextrin (β-CD) [65]. The researchers also tested 2,6-di-*O*-methyl-β-cyclodextrin (DM-β-CD) and examined its ability to enhance water solubility and improve in vitro dissolution. To form the inclusion complexes, β-CD and DM-β-CD were dissolved in 40% ethanol at a 1:1 molar ratio with CBD. The solutions were combined, filtered, and then lyophilized in a vacuum freeze dryer. Both inclusion complexes achieved high encapsulation efficiency: β-CD (92.4 ± 0.49%) and DM-β-CD (90.8 ± 0.34%), indicating effective incorporation of CBD into the cyclodextrin cavities. The loading efficiency for β-CD was 20.4 ± 0.53%, while DM-β-CD was slightly lower at 17.7 ± 0.69%. Regarding API solubility, β-CD and DM-β-CD achieved significantly higher CBD dissolution rates than raw CBD. β-CD resulted in a dissolution of 0.395 µg/mL, while DM-β-CD achieved a remarkable 14.118 µg/mL, compared to just 0.023 µg/mL for raw CBD. This represents a 17-fold and 614-fold increase in solubility, respectively. The in vitro dissolution study further confirmed that the inclusion complexes enhanced the dissolution rate of CBD. The formulation with DM-β-CD exhibited the most significant improvement, releasing 66.77% of the contained CBD within 30 min and almost all after 240 min. In contrast, the β-CD formulation released only 1.9% after 30 min, reaching a maximum of 2.8% after 180 min. The authors attributed the improved solubility and faster dissolution to the good solubility of DM-β-CD in water. The ability of many water molecules to enter the cyclodextrin cavity helps displace the API, while the swelling of the cyclodextrin structure may also contribute to this rapid release [65].

Although these preliminary studies show that cyclodextrin inclusion complexes could effectively improve CBD bioavailability, the field is still in its initial stages. Ongoing research on various cyclodextrin derivatives, optimization of formulation methods, and comprehensive in vivo studies may provide valuable insights into the full therapeutic potential of CBD, making this an exciting and underexplored area in pharmaceutical science.

## 6. Comparative Synthesis and Ranking

The comparative analysis of advanced nanocarrier systems for CBD highlights both their potential and their translational challenges. Table 3 summarizes pharmacokinetic outcomes of representative systems in animal and human studies. SNEDDSs demonstrate the most pronounced improvement in oral bioavailability (up to 7–14-fold increases in AUC compared to oils or powders), while polymeric nanoparticles, oleosomes, and other carriers show moderate but consistent gains. These differences underscore the variability in systemic exposure depending on carrier design and route of administration.

**Figure 1:**
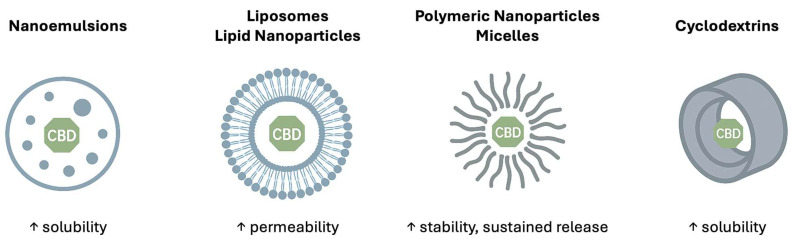
Schematic representation of the main nanocarriers used for cannabidiol (CBD) delivery. The up arrow indicates growth.

To better explain these outcomes, Table 4 and Figure 1 outline the key mechanisms by which each delivery system enhances bioavailability. For example, SNEDDSs and nanoemulsions act by increasing solubility and dissolution rate in the gastrointestinal tract, while liposomes improve membrane permeability and protect CBD from degradation. Polymeric nanoparticles provide controlled release and stability, nanosuspensions accelerate dissolution through particle size reduction, cyclodextrin complexes improve aqueous solubility, and oleosomes uniquely promote lymphatic absorption.

Finally, Table 5 integrates these findings into a comparative ranking of platforms by clinical readiness, safety, and scalability. SNEDDSs emerge as the most translation-ready, with early human evidence and industrial know-how, though safety concerns related to high surfactant levels remain. Liposomes, polymeric nanoparticles, and nanosuspensions are promising but face challenges of manufacturing complexity, stability, or limited clinical validation. Cyclodextrins and oleosomes, while simple or naturally derived, remain at a preliminary stage with sparse in vivo or clinical data.

Collectively, this synthesis reveals that while SNEDDSs are currently closest to clinical translation, no single platform provides a complete solution. Each technology offers distinct strengths (e.g., rapid onset, controlled release, lymphatic targeting) but also notable limitations (e.g., metabolic interactions, instability, or regulatory barriers). This highlights the need for hybrid and smart delivery systems that combine complementary advantages.

## 7. Future Perspective

The field of CBD bioavailability enhancement through nanotechnology and advanced delivery systems is rapidly evolving. Despite progress with nanoemulsions, liposomes, polymeric micelles, nanosuspensions, and cyclodextrin complexes, significant challenges remain in optimizing formulations, ensuring long-term safety, and achieving regulatory approval.

Among the various delivery systems investigated, SNEDDSs currently demonstrate the strongest translational potential, supported by both preclinical and clinical data. Future research should focus on confirming their long-term safety, optimizing solid SNEDDS formulations for improved stability, and directly comparing their performance with alternative carriers such as liposomes, oleosomes, and polymeric nanoparticles. Expanding well-designed clinical trials will be essential to determine which platform can most effectively translate into safe and standardized CBD therapies.

A key direction for future work is the development of hybrid systems that integrate complementary technologies, for example, combining SNEDDSs with polymeric nanoparticles or cyclodextrins, to achieve synergistic improvements in solubility, permeability, and stability. In addition, smart nanocarriers responsive to physiological cues (e.g., pH, enzymes, or temperature) could enable programmable, site-specific CBD release while minimizing systemic exposure and side effects. Another promising approach involves the use of natural bioenhancers (e.g., piperine, curcumin, flavonoids), which may reduce first-pass metabolism and increase systemic exposure when co-administered with CBD. Personalized formulations tailored to pharmacogenomic profiles could further optimize efficacy and safety, especially in chronic conditions such as epilepsy, anxiety, and neurodegenerative disorders.

### 7.1. Manufacturing and Regulatory Challenges

Large-scale manufacturing of CBD nanocarriers remains challenging, often requiring costly equipment, complex processes, or excipients that are difficult to standardize. These factors limit scalability and reproducibility. From a regulatory perspective, robust data on safety, stability, and batch-to-batch consistency are essential, alongside long-term toxicology studies for novel excipients. Currently, no CBD nanoformulation has gained regulatory approval, although SNEDDSs appear closest to translation, supported by human pharmacokinetic evidence of superior absorption compared with conventional oils.

### 7.2. Safety, Toxicity, and Clinical Translation

While nanocarriers offer promising solutions to overcome CBD’s bioavailability limitations, their long-term safety remains a critical concern. Many formulations employ high concentrations of surfactants, polymers, or penetration enhancers that may cause gastrointestinal irritation, cytotoxicity, or systemic adverse effects during chronic use. For example, SNEDDSs often require surfactant levels above regulatory thresholds, raising tolerability issues [34]. Liposomes and polymeric nanoparticles, although biocompatible in principle, are prone to lipid peroxidation, polymer degradation, or excipient-related immunogenicity [66]. Additionally, some advanced systems such as Pro-Nanolipospheres intentionally inhibit metabolic enzymes to enhance absorption [67], but this introduces risks of significant drug–drug interactions and toxic accumulation of co-administered medications. Cyclodextrins, while generally recognized as safe, may alter gut microbiota metabolism or cause renal stress at high doses [68]. Regulatory approval is further complicated by the lack of standardized toxicological testing protocols for nanocarriers [69]. Few studies provide systematic evaluation of genotoxicity, immunogenicity, or chronic organ-specific toxicity. Moreover, most data available are limited to animal or in vitro models, and human safety evidence remains scarce. Addressing these concerns will require comprehensive preclinical toxicology, long-term safety studies, and robust pharmacovigilance frameworks during clinical translation. Only by balancing efficacy with safety and regulatory compliance can CBD nanocarriers advance toward standardized and reliable therapeutic use. So, future research should prioritize comprehensive toxicity testing, including long-term studies on excipients and cumulative safety during chronic administration. Standardized protocols for toxicology, immunogenicity, and organ-specific evaluation will be crucial to support regulatory approval. In parallel, early pharmacovigilance strategies should be established as formulations enter clinical trials, with a focus on adverse event monitoring, drug–drug interactions, and post-marketing surveillance. Establishing such frameworks will be essential to ensure that improvements in bioavailability translate into safe, scalable, and clinically viable CBD therapies.

## 8. Conclusions

Nanotechnology and advanced delivery systems offer powerful strategies to overcome the poor bioavailability of cannabidiol (CBD). Among the various platforms, self-nanoemulsifying drug delivery systems (SNEDDSs) currently show the strongest translational potential, supported by both preclinical and early clinical evidence. Hybrid nanocarriers, integration with natural bioenhancers, and personalized formulations tailored to patient profiles represent particularly promising directions for future development.

Nevertheless, key challenges remain, including long-term safety, toxicity, large-scale manufacturing, and regulatory approval. Addressing these barriers through systematic toxicological testing, well-designed clinical trials, and standardized production protocols will be essential for successful translation.

It is also interesting to consider the impact of other cannabinoids on the stability, safety, and systemic pharmacological action of CBD obtained from natural sources. The “entrance” phenomenon is well-defined in relation to profiling and personalizing CBD treatments, but it remains a topic of ongoing research in the development of nano-scale CBD delivery systems. It is also worth noting the impact of so-called related cannabinoids on the bioavailability of CBD, even when administered via nano-scale delivery systems.

Future progress will depend not only on improving bioavailability but also on ensuring safety, scalability, and regulatory compliance, critical steps to transform CBD nanocarriers from experimental systems into clinically viable therapeutics.

## Figures and Tables

**Table 1:** Summary of possible molecular targets of CBD, effects of their action, and potential for application.

Receptor Type	Action	Effects of Action	Possible Applications	Refs.
CB_1_	Antagonistic modulator	Anticonvulsant effect	Epilepsy	[15]
GPR_12_	Inverse agonist	Promotes neurite outgrowth and blocks myelin inhibition in neurons	Neurodegenerative diseases	[16]
GPR_55_	Antagonist	Anti-inflammatory effect, Analgesic effect	Neurodegenerative diseases, Analgesia/Coanalgesia	[17]
GlyR_α1_	Allosteric modulator	Antiepileptic effect	Epilepsy	[18]
GlyR_α1β_	Allosteric modulator	Antiepileptic effect	Epilepsy	[18]
GlyR_α3_	Potentiator	Analgesic effect	Neuropathic pain	[19]
5-HT_1A_	Agonist	Antiepileptic effect, Anticataleptic effect, Antipsychotic effect	Epilepsy, Neuroleptic malignant syndrome, Anxiety	[20,21]
5-HT_2A_	Agonist	Antiepileptic effect, Anticataleptic effect	Epilepsy, Neuroleptic malignant syndrome,	[20,21]
5-HT_3A_	Antagonist	Antiemetic	Vomiting associated with cancer treatment	[22]
PPARγ	Agonist	Antitumor effect, Anti-inflammatory effect	Neurodegenerative diseases	[23,24]
AR_A1_	Agonist	Antiarrhythmic effect, Anti-inflammatory effect, Antipsychotic effect	Ventricular tachycardia, Neurodegenerative diseases, Anxiety	[25,26,27]

**Table 2:** IC_50_ values (μg/)mL of free CBD, CP-liposome, and GMCP-liposome against different tumor cell lines after 72 h incubation [45].

		Cell Line					
		4T1	MCF-1	A549	C6	Hela	HepG2
Fold of increased permeability	CP-Liposomes	36.3	8.6	2.2	1.8	4.3	3.2
	GMCP-Liposomes	19.4	6.5	2.2	2.2	3.5	3.5

**Table 3:** Pharmacokinetic performance of CBD delivery systems after oral administration. The up arrow indicates growth.

Delivery System	Species/Model	C_max_ (ng/mL)	AUC_0–∞_ (ng·h/mL)	Relative Bioavailability	Ref.
SNNEDSs	Human (healthy volunteers)	18 ± 9 _L-SNNEDS_	66 ± 19 _L-SNNEDS_	~7.0-fold ↑ (vs. powder)	[38]
	Rat	499.5 ± 144.6 _L-SNNEDS_ 283.7 ± 84.7 _S-SNNEDS-SD_ 146.7 ± 26.0 _S-SNEEDS-SCA_	1534.3 ± 438.9 _L-SNNEDS_ 833.8 ± 214.7 _S-SNNEDS_SD_ 642.5 ± 77.4 _S-SNEEDS-SCA_	~6–14-fold ↑ (vs. MCT oil)	[36]
Polymeric nanoparticles	Rat	0.466 ± 0.023	2.912 ± 0.310	~1.75-fold ↑ (vs. pure CBD)	[59]
Oleosomes	Rat	79 ± 11	413 ± 25	~1.5-fold ↑ (vs. oil)	[52]

**Table 4:** Mechanisms by which different nanocarrier systems enhance CBD bioavailability. The up arrow indicates growth.

Nanocarrier System	Mechanism of Action	Effect on Bioavailability (PK Outcome)
SNEDDSs/Nanoemulsions	Spontaneous formation of nano-sized emulsions in GI tract → ↑ surface area, partial lymphatic transport	↑ Solubility, ↑ dissolution rate, ↑ oral absorption, partial bypass of first-pass metabolism
Liposomes/Transfersomes	Phospholipid vesicles protect CBD; enhanced membrane fluidity and skin/mucosal penetration	Controlled release, protection from degradation, ↑ permeability, potential for transdermal/oral delivery
Polymeric NPs/Micelles	Encapsulation in biodegradable polymer matrix; controlled degradation and drug release	↑ Stability, sustained release → ↑ AUC, potential for tissue targeting
Nanosuspensions	Size reduction to nanocrystals → ↑ surface area for dissolution	Rapid dissolution, ↑ C_max_, possibility of parenteral administration
Cyclodextrin complexes	Inclusion of CBD in hydrophobic cavity of cyclodextrin → ↑ aqueous solubility	↑ Solubility and dissolution, moderate ↑ oral bioavailability
Oleosomes	Natural lipid bodies stabilize CBD; promote lymphatic absorption	Protection from degradation, lymphatic transport → partial bypass of first-pass metabolism, ↑ systemic exposure

**Table 5:** Comparative synthesis of CBD nanocarrier platforms ranked by clinical readiness, safety data, and scalability.

Platform	Clinical Readiness	Safety Data Depth	Scalability/Manufacturability	Key Strengths	Main Limitations
SNEDDS	High (human PK evidence; closest to translation)	Medium (tolerability events; surfactant load; P-PNL DDI risk)	Medium–High (oil/surfactant systems; solidification adds complexity)	Big oral exposure gains; formulation know-how	First-pass not fully overcome; chronic safety of excipients; DDI with enzyme inhibition
Liposomes	Medium–Low (mainly preclinical/transdermal)	Medium (biocompatible lipids but oxidation/irritancy risks)	Low–Medium (stability control and cost)	Controlled release; membrane crossing; skin delivery	Chemical/physical instability; cost and scale-up hurdles; limited clinical data
Polymeric NPs	Medium–Low (enhanced effects vs. free CBD, limited clinical)	Medium (novel excipients; biodegradability/chronic-use questions)	Low–Medium (solvents/complex processes)	Stability; tunable release; targeting potential	Regulatory burden for new polymers; manufacturing complexity; burst release
Nanosuspensions	Medium–Low (animal PK ↑; parenteral option)	Medium (well-known excipients; needs chronic data)	Medium (unit ops established; CBD-specific know-how evolving)	High drug load; solvent-lean; fast dissolution	Limited clinical data; route/formulation optimization needed
Cyclodextrin complexes	Low (early stage; in vitro dissolution focus)	Medium (GRAS history but dose-related concerns)	High (simple processing; excipient supply)	Huge solubility/dissolution boosts	Sparse in vivo efficacy; dose-limit safety considerations
Oleosomes	Low (promising animal PK and lymphatic transport)	Medium (natural carriers; standardization pending)	Low–Medium (source variability; standardization challenges)	Natural, stable emulsions; strong lymphatic targeting	Reproducibility/regulatory acceptance; no clinical data yet

High = human data and near-term manufacturability with a manageable safety profile; Medium = encouraging preclinical, partial human or clear manufacturability path; Low = early/preclinical with notable gaps. Safety = breadth/quality of toxicology, human tolerability data. Scalability = process simplicity, excipient availability, and industrial precedent. The up arrow indicates growth.

## Data Availability

No new data were created or analyzed in this study. Data sharing is not applicable to this article.

## Abbreviations

The following abbreviations are used in this manuscript:

CBD	Cannabidiol
THC	Δ^9^-tetrahydrocannabinol
GPCR	G-Protein-Coupled Cannabinoid CB1 Receptor
GlyRα1/α2/β	Glycine Receptor alpha-1, alpha-3, beta
5-HT1A/2A/3A	5-hydroxytryptamine Receptor 1A, 2A, 3A
PPARγ	Peroxisome Proliferator-Activated Receptor Gamma
ARA1	Adenosine A1 Receptor
FDA	U.S. Food and Drug Administration
EMA	European Medicines Agency
CBD-NE	Cannabidiol contained Nanoemulsion
SNEDDS	Self-Nanoemulsifying Drug Delivery System
AUC	Area Under the Curve
C_max_	Maximum Serum Concentration
PNLs	Pro-Nanolipospheres
MLVs	Multilamellar Vesicles
MVVs	Multivesicular Vesicles
GUVs	Giant Unilamellar Vesicles
LUVs	Large Unilamellar Vesicles
SUVs	Small Unilamellar Vesicles
SPC	Soybean Phosphatidylcholine
PEG 400	Polyethylene Glycol 400
PPD	20(S)-Protopanaxadiol
CP-liposomes	Cannabidiol and 20(S)-Protopanaxadiol contained liposomes
GMCP-liposomes	Cannabidiol, 20(S)-Protopanaxadiol and *n*-Dodecyl β-D-maltoside contained liposomes
PBS	Phosphate-Buffered Saline
HPMCAS	Hydroxypropylmethylcellulose Acetate Succinate
FD	Ester of Fucoidan and Deoxycholic Acid
API	Active Pharmaceutical Ingredient
PLGA	Poly-lactic-*co*-glycolic acid
NPs	Nanoparticles
CAM	Chorioallantoic Membrane
WP	Whey Protein
β-CD	β-Cyclodextrin
DM-β-CD	2,6-di-*O*-methyl-β-cyclodextrin
